# Innate biology versus lifestyle behaviour in the aetiology of obesity and type 2 diabetes: the GLACIER Study

**DOI:** 10.1007/s00125-015-3818-y

**Published:** 2015-12-01

**Authors:** Alaitz Poveda, Robert W. Koivula, Shafqat Ahmad, Inês Barroso, Göran Hallmans, Ingegerd Johansson, Frida Renström, Paul W. Franks

**Affiliations:** Genetic and Molecular Epidemiology Unit, Department of Clinical Sciences, Lund University, Clinical Research Center Building 91, Level 10, Jan Waldenströms gata 35, SE-20502 Malmö, Sweden; Department of Genetics, Physical Anthropology and Animal Physiology, Faculty of Science and Technology, University of the Basque Country (UPV/EHU), Bilbao, Spain; Wellcome Trust Sanger Institute, Wellcome Trust Genome Campus, Hinxton, Cambridge, UK; NIHR Cambridge Biomedical Research Centre, Institute of Metabolic Science, Addenbrooke’s Hospital, Cambridge, UK; Metabolic Research Laboratories Institute of Metabolic Science, University of Cambridge, Addenbrooke’s Hospital, Cambridge, UK; Department of Biobank Research, Umeå University, Umeå, Sweden; Department of Odontology, Umeå University, Umeå, Sweden; Department of Public Health and Clinical Medicine, Section for Medicine, Umeå University, Umeå, Sweden; Department of Nutrition, Harvard Chan School of Public Health, Boston, MA USA

**Keywords:** Environment, Genes, Obesity, Predictors, Type 2 diabetes

## Abstract

**Aims/hypothesis:**

We compared the ability of genetic (established type 2 diabetes, fasting glucose, 2 h glucose and obesity variants) and modifiable lifestyle (diet, physical activity, smoking, alcohol and education) risk factors to predict incident type 2 diabetes and obesity in a population-based prospective cohort of 3,444 Swedish adults studied sequentially at baseline and 10 years later.

**Methods:**

Multivariable logistic regression analyses were used to assess the predictive ability of genetic and lifestyle risk factors on incident obesity and type 2 diabetes by calculating the AUC.

**Results:**

The predictive accuracy of lifestyle risk factors was similar to that yielded by genetic information for incident type 2 diabetes (AUC 75% and 74%, respectively) and obesity (AUC 68% and 73%, respectively) in models adjusted for age, age^2^ and sex. The addition of genetic information to the lifestyle model significantly improved the prediction of type 2 diabetes (AUC 80%; *p* = 0.0003) and obesity (AUC 79%; *p* < 0.0001) and resulted in a net reclassification improvement of 58% for type 2 diabetes and 64% for obesity.

**Conclusions/interpretation:**

These findings illustrate that lifestyle and genetic information separately provide a similarly high degree of long-range predictive accuracy for obesity and type 2 diabetes.

**Electronic supplementary material:**

The online version of this article (doi:10.1007/s00125-015-3818-y) contains peer-reviewed but unedited supplementary material, which is available to authorised users.

## Introduction

Type 2 diabetes and obesity are caused by three forces: genetic predisposition, the action of environmental factors and interaction between the two. While genetic factors may predispose a person to type 2 diabetes or obesity, a permissive environment can promote the development of these cardiometabolic diseases.

In recent work by the Genetic Investigation of Anthropometric Traits (GIANT) consortium [1], almost 100 loci were reproducibly associated with adult BMI. Two other consortia (Diabetes Genetics Replication and Meta-Analysis [DIAGRAM] and the Meta-Analyses of Glucose and Insulin-Related Traits Consortium [MAGIC]) have also identified genetic variants associated with type 2 diabetes [2] and fasting and 2 h glucose concentrations [3]. However, these loci account for only a small fraction of the estimated genetic variation in BMI (∼2.7%) and type 2 diabetes (∼5.7%) [1, 2].

Although several studies have compared the ability of genetic and clinical risk scores to predict type 2 diabetes [4–6], quantitative phenotypes (e.g. blood glucose or cholesterol) used in these clinical risk scores are biological intermediates of disease, resulting from the combination of both unfavourable lifestyle and genotype. To our knowledge, no published prospective studies have reported on head-to-head comparisons of the full array of established genetic variants and lifestyle risk factors for type 2 diabetes and obesity. The current study sought to address this knowledge gap in a population-based prospective cohort of Swedish adults, the Gene–Lifestyle Interactions and Complex Traits Involved in Elevated Disease Risk (GLACIER) Study [7].

## Methods

### Study participants

The GLACIER Study is a prospective, population-based cohort study nested within the Northern Sweden Health and Disease Study (NSHDS) [7, 8]. Lifestyle and clinical data were collected within the framework of the Västerbottens Hälsoundersökning (Västerbotten Health Survey [VHU]) initiated in 1985, inviting all residents within the county to attend an extensive health examination in the years of their 40th, 50th and 60th birthdays. Thus, the vast majority of participants had follow-up examinations roughly 10 years after baseline. However, seven participants underwent follow-up examinations between four and six years after baseline. Initially, residents turning 30 years of age were also invited but this was later dropped. Of the 5,726 GLACIER participants with necessary genotypic and phenotypic information, 3,444 had follow-up data available. Baseline examinations were performed between 1990 and 1999, and follow-up examinations between 1995 and 2008. All participants provided written informed consent as part of the VHU, and the study was approved by the Regional Ethical Review Board in Umeå, Sweden.

### Clinical measures

The assessment of clinical measures has been described in detail elsewhere [7]. Briefly, weight (to the nearest 0.1 kg) and height (to the nearest 1 cm) were measured with a calibrated balance-beam scale and a wall-mounted stadiometer, respectively, with participants wearing indoor clothing and without shoes. Normal weight was defined as BMI of 18.5–24.9 kg/m^2^, overweight as BMI 25–29.9 kg/m^2^ and obesity as BMI ≥30 kg/m^2^. Capillary blood was drawn after an overnight fast, and a second sample was drawn 2 h after a standard 75 g oral glucose load [9]. Capillary plasma glucose concentrations were measured with a Reflotron bench-top analyser (Roche Diagnostics Scandinavia, Umeå, Sweden).

At baseline and follow-up, 78% and 98% of the participants reported having fasted for a minimum of 8 h, respectively. A variable was, therefore, included in the analysis with glycaemic traits to control for fasting time. Type 2 diabetes was determined based on self-report or from a 75 g oral glucose tolerance test performed as part of the VHU. Type 2 diabetes was defined according to the criteria of the American Diabetes Association [10] as a fasting plasma glucose concentration ≥7.0 mmol/l or a 2 h plasma glucose concentration ≥11.1 mmol/l. Fasting glucose concentration was categorised into three levels: normal fasting glucose (<6.1 mmol/l), impaired fasting glucose (IFG; between ≥6.1 and <7.0 mmol/l) and diabetic fasting glucose (≥7.0 mmol/l). Two hour glucose concentration was categorised into three levels: normal glucose tolerance (<7.8 mmol/l), impaired glucose tolerance (IGT; between ≥7.8 and <11.1 mmol/l) and diabetic glucose tolerance (≥11.1 mmol/l). Incident cases of IFG and IGT were defined as participants changing from normal to impaired status during follow-up.

### Genotyping

DNA was extracted from peripheral white blood cells, and genomic DNA samples were diluted to 4 ng/μl as previously described [11, 12]. Genotyping was performed using the Metabochip array (Illumina, San Diego, CA, USA) [13] at the Wellcome Trust Sanger Institute, UK. Ninety-seven BMI-associated loci [1], 65 type 2 diabetes-associated loci [2], 36 fasting glucose loci and nine 2 h glucose-associated loci [3] were extracted. Genotypes were coded as 0, 1 and 2 at each single nucleotide polymorphism (SNP) locus, indicating the number of effect alleles (as defined by the prior meta-analyses [1–3]) per participant. We used proxy SNPs for 26 loci, as indicated in electronic supplementary material (ESM) Tables 1 and 2. Missing rate was ≤0.07 per participant and ≤0.007 per SNP. Missing genotypes were imputed using mean imputation as previously described [14] by replacing each missing genotype with its mean value, which was obtained from the fraction of the cohort having genotype data available. No significant deviations from Hardy–Weinberg equilibrium (*p* < 0.0001) were observed.

#### Genetic risk scores

In order to examine the cumulative effects of the SNPs, four genetic risk scores (GRS) were generated for each participant by summing the number of effect alleles at each associated SNP for: (1) obesity (ob-GRS); (2) fasting glucose (fg-GRS); (3) 2 h glucose (2hg-GRS); and (4) type 2 diabetes (t2d-GRS). The minimum theoretical value of all four GRS is 0 and the maximum theoretical values are 194 for ob-GRS (range 70–114), 72 for fg-GRS (range 27–50), 18 for 2hg-GRS (range 2–13) and 130 for t2d-GRS (range 50–89).

### Lifestyle assessment

Diet was assessed using a validated semi-quantitative food frequency questionnaire (FFQ) designed to capture habitual diet over the last year [15–17]. Participants indicated how often they consumed foods and beverages on a nine-point frequency scale, ranging from never to four or more per day, and also indicated average portion size of meat and fish, vegetables, potatoes, rice and pasta. Total energy intake was calculated based on the National Food Administration database (www.slv.se). The initial FFQ (used from 1985) covered 84 food items, but in 1996 was reduced to 66 food items by combining several questions related to similar foods. All analyses including dietary variables were adjusted for a variable indicating FFQ version. The current analysis included intakes of total energy (kcal/day), alcohol (g/day), salt (g/day), sucrose (g/day), macronutrients (g/day; carbohydrate, protein, total fat, saturated fat, monounsaturated fatty acids [MUFA], polyunsaturated fatty acids [PUFA], essential fatty acids [*n*-3 and *n*-6 fatty acids] and fibre), vitamins and minerals (vitamins A [mg/day], D [μg/day], E [mg/day], B6 [mg/day], B12 [μg/day] and C [mg/day], thiamin [mg/day], riboflavin [mg/day], niacin [mg/day], folate [μg/day], calcium [mg/day], phosphorus [mg/day], potassium [mg/day], magnesium [mg/day], iron [mg/day], zinc [mg/day], iodine [μg/day] and selenium [μg/day]). Participants with ≥10% of the FFQ missing or an implausible total energy intake (<500 or >4500 kcal/day; <2093 or >18841 kJ/day) were excluded from the analyses.

Apart from diet variables, the lifestyle factors used were smoking status (current smokers, ex-smokers, non-smokers), education (school, college and university levels) and physical activity. Physical activity was assessed through a modified version of the International Physical Activity Questionnaire [18, 19], which gathers information on leisure time physical activity for the past 3 months categorised as never, occasionally, 1–2 times/week, 2–3 times/week or >3 times/week. For the current analysis, categories were combined into physically inactive (never and occasionally) and physically active (≥1–2 times/week).

#### Diet scores

Three diet scores were constructed and tested for association with incidence of obesity and type 2 diabetes. The Healthy Diet (HD) score was constructed from intakes of eight food groups: whole grains, fish, fruits and vegetables were designated as favourable foods, whereas red and processed meats, desserts and sweets, sugar-sweetened beverages and fried potatoes were designated as unfavourable. The original HD score additionally includes nuts but this information is not available in the VHU. Intake of each food group was categorised into quartiles, and ascending values (0,1,2,3) were assigned for favourable foods and descending values (3,2,1,0) for unfavourable foods. These values were then summed to generate the HD score, with higher scores indicating a healthier diet [20].

The second score, the Nordic nutrition recommendation (NNR) score, was constructed following the recommendations of the Nordic Council of Ministers [21]. For each recommendation, 1 point was assigned when the recommendation was fulfilled and 0 points when the recommendation was not fulfilled. The points for each participant were subsequently summed, with a higher score indicating a healthier diet. A full description of the recommendations used to construct the NNR score is given in ESM Table 3.

A third score was constructed by conducting a principal component analysis (PCA) in order to obtain a summary factor representing global dietary intake. All of the macronutrients analysed in this study were included in the analysis and the model was adjusted for total energy intake. A single factor was retained that contrasted carbohydrate and fibre intake against fat intake and accounted for 54% of the variance of all macronutrients (ESM Table 4). Spearman’s correlations between the three diet scores (partialled for age, age^2^, sex, FFQ version and total energy intake) were calculated. All diet scores were significantly correlated with each other (*p* < 0.0001); NNR score and HD score were positively correlated (*r* = 0.36), and both scores were negatively correlated with the PCA score (*r* = −0.21 and −0.33, respectively).

### Statistical analyses

After excluding participants who were classified with obesity or type 2 diabetes at baseline, the predictive ability of genetic and lifestyle risk factors in incident obesity and type 2 diabetes during the 10 year follow-up period was assessed by logistic regression analysis. Multivariable logistic regression was also used to predict weight gain ≥10%, and incident IFG and IGT during follow-up.

In the prospective analyses, three different models were used: Model 1 (the genetic model) included age, age^2^, follow-up duration, fasting status (for glycaemic traits), sex and trait-specific SNPs as independent variables; Model 2 (the lifestyle model) included age, age^2^, follow-up duration, fasting status (for glycaemic traits), sex, FFQ version, education, smoking status, physical activity and intakes of total energy, alcohol, salt, sucrose, macronutrients, vitamins and minerals; Model 3 (the combined model) included all variables in Models 1 and 2 above.

Age and sex were included in all models as both are strong predictors of type 2 diabetes and obesity, and excluding them from lifestyle and/or genetic models may cause bias and confounding. This is, in part, because age and sex are stronger confounders of the lifestyle effect estimates compared with the genetic effect estimates in our analyses. Thus, without adjustment, the comparison of genetic and lifestyle models is likely to be biased by the greater degree of confounding in the latter than in the former models. BMI, which is a strong predictor of type 2 diabetes, was not included in glycaemic trait models as it carries combined information on both lifestyle and genetic risk factors and including it in either model (genetic or lifestyle) could unduly influence the model.

#### Predictive ability

The predictive accuracy of the models outlined above was assessed by calculating the AUC. The AUCs of the different models were compared using the method described by DeLong et al [22]. The sensitivity of the models at 90% specificity was also estimated.

#### Net reclassification improvement

The continuous net reclassification improvement (cNRI), which quantifies the correctness of upward and downward reclassification as a result of adding the genetic information to the lifestyle model, was calculated [23].

#### Model calibration

Model calibration was assessed by Akaike’s information criterion (AIC) and the Hosmer–Lemeshow test [24].

#### Association of genetic and lifestyle factors

For models focused on predictive accuracy, lifestyle variables and genetic variants were entered individually to improve predictive power (see above). However, for association analyses, diet scores and GRS were calculated to obtain an overall estimate of the effect of diet and genetic information. In addition, although multicollinearity does not affect the predictive power of the models, it affects estimates of the individual predictors [25]. By constructing GRS and diet scores, multicollinearity was avoided when calculating ORs. In order to investigate how modest differences in lifestyle and genetic factors related to type 2 diabetes and obesity risk, the quartiles of each diet and genetic score were calculated. As alcohol intake was not part of any of the diet scores, quartiles of this variable were calculated and included in the models. The top and the bottom quartiles of each score and lifestyle variable were compared in the models.

#### Multi-trait genetic information

Type 2 diabetes is a heterogeneous phenotype that probably includes several subtypes of diabetes, and genetic variants operating through different pathways (e.g. obesity) may have greater predictive value for diabetes or its subtypes than others [26]. Thus, we also evaluated the predictive ability on incident type 2 diabetes for all variants associated with type 2 diabetes, fasting and 2 h glucose, and obesity. SNPs associated with multiple traits or in linkage disequilibrium (LD; *r*^2^ > 0.8) were included only once in the model.

Data were analysed with PLINK (version 1.07) [27], R (version 3.1.1) [28] and SAS (version 9.4, SAS Institute, Cary, NC, USA) [29].

## Results

Baseline participant characteristics and incidence of obesity, type 2 diabetes, IFG and IGT are summarised in Table 1 and ESM Table 5. Of the 3,444 participants followed for a median of 9.9 ± 0.4 years, 264 (7.7%) developed obesity, 192 (5.6%) type 2 diabetes, 563 (16.3%) IFG and 613 (17.8%) IGT.

**Table 1:** Incidence of obesity, type 2 diabetes, IFG and IGT and baseline descriptive characteristics of the GLACIER Study participants with follow-up information

Variables	Men	Women	All
	(*n* = 1,322)	(*n* = 2,122)	(*n* = 3,444)
Age, years	45.8 ± 6.5	44.9 ± 6.8	45.2 ± 6.7
Length of follow-up, years	10 ± 0.4	9.9 ± 0.4	9.9 ± 0.4
Fasting status at baseline, % missing/<4 h/4–8 h/>8 h	19.3/0.9/5.0/74.8	15.1/0.6/5.1/79.2	16.7/0.7/5.0/77.6
Fasting status at follow-up, % missing/<4 h/4–8 h/>8 h	1.1/0/1.5/97.4	1.0/0/0.6/98.4	1.1/0/0.9/98.0
Incident obesity, *n* (%)	105 (7.9)	159 (7.5)	264 (7.7)
Incident type 2 diabetes, *n* (%)	98 (7.4)	94 (4.4)	192 (5.6)
Incident IFG, *n* (%)	232 (17.5)	331 (15.6)	563 (16.3)
Incident IGT, *n* (%)	211 (16.0)	402 (18.9)	613 (17.8)
BMI, kg/m^2^	25.6 ± 3.3	24.8 ± 4.0	25.1 ± 3.7
Fasting glucose, mmol/l	5.4 ± 0.7	5.3 ± 0.6	5.3 ± 0.7
2 h glucose, mmol/l	6.2 ± 1.5	6.7 ± 1.4	6.5 ± 1.4

Data are expressed as mean ± SD for quantitative variables and as *n* and/or percentage for qualitative variables

### Obesity

#### Predictive ability

**Fig. 1:**
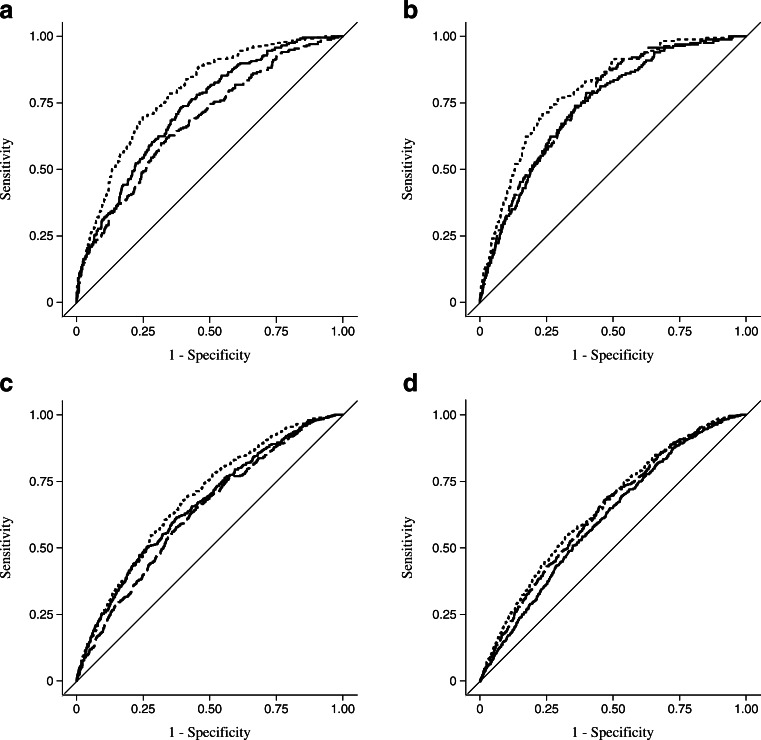
AUCs for incidence of (**a**) obesity, (**b**) type 2 diabetes, (**c**) IFG and (**d**) IGT. Solid line, genetic model; dashed line, lifestyle model; dotted line, combined model

The lifestyle model had a similar predictive accuracy of incident obesity as the genetic model (AUC 68% vs 73%; *p*_difference_ = 0.08; Table 2). The predictive ability of these models was lower when the models were not adjusted for age and sex (AUC 67% and 72% for lifestyle and genetic models, respectively). The addition of genetic information (97 SNPs) to the lifestyle model significantly improved the predictive ability of the model (AUC 79%; *p*_difference_ < 0.0001; Fig. 1a). Sensitivity of the combined model was maximised (38%) at a fixed specificity of 90%. Sensitivity was higher in the model that included only the genetic factors compared with the model that included only lifestyle factors (31% vs 26%).

**Table 2:** Predictive ability and model calibration for prediction of incident obesity (*n* = 1,511), type 2 diabetes (*n* = 2,017), IFG (*n* = 2,778) and IGT (*n* = 2,420) based on lifestyle and genetic factors alone and in combination

Trait	Model	AUC (95% CI)	AUC *p* value^a^	Sensitivity (90% specificity)	AIC	Hosmer–Lemeshow *p* value
Obesity	Genetic model	0.726 (0.693, 0.759)	0.08	31%	1,377	0.56
	Lifestyle model	0.681 (0.644, 0.718)		26%	1,301	0.57
	Combined model	0.789 (0.759, 0.818)	<0.0001	38%	1,362	0.52
Type 2 diabetes	Genetic model	0.740 (0.704, 0.777)	0.47	32%	1,176	0.64
	Lifestyle model	0.754 (0.720, 0.789)		33%	1,107	0.30
	Combined model	0.797 (0.765, 0.830)	0.0003	40%	1,179	0.57
IFG	Genetic model	0.662 (0.636, 0.688)	0.05	25%	2,538	0.22
	Lifestyle model	0.633 (0.607, 0.660)		20%	2,583	0.45
	Combined model	0.686 (0.661, 0.711)	<0.0001	26%	2,570	0.25
IGT	Genetic model	0.612 (0.586, 0.637)	0.03	16%	2,557	0.92
	Lifestyle model	0.640 (0.614, 0.665)		19%	2,574	0.83
	Combined model	0.651 (0.626, 0.677)	0.03	22%	2,577	0.32

Sample sizes are comprised of participants who experienced an event during follow-up (i.e. participants who were free of obesity, type 2 diabetes, IFG or IGT at baseline and had developed the respective disease/condition at follow-up) and healthy controls (i.e. those who were healthy at baseline and at follow-up) with relevant lifestyle, genetic and demographic data. All other participants from the overall cohort were excluded from these analyses

^a^AUC *p* values are for genetic and combined models vs lifestyle model

Concerning weight gain ≥10%, genetic and lifestyle models showed a predictive ability of 65% and the addition of the genetic information to the lifestyle model significantly improved its predictive ability (AUC 68%; *p*_difference_ = 0.0004; ESM Table 6).

#### Net reclassification improvement

The addition of genetic information to the lifestyle factors resulted in a cNRI of 64% (*p* < 0.0001; Table 3), comprised of an event cNRI of 32% and a non-event cNRI of 32%. The addition of genetic information to the lifestyle model also improved the predictive ability of the model for weight gain ≥10% (cNRI 26%; *p* < 0.0001; ESM Table 6) and the effect of the reclassification was higher for participants with <10% weight gain during follow-up (cNRI 11% for event vs 15% for non-event).

**Table 3:** cNRI based on the addition of genetic information to lifestyle variables

Trait	cNRI (95% CI)	*p* value	cNRI event	cNRI non-event
Obesity, %	64.04 (50.92, 77.16)	<0.0001	32.49	31.55
Type 2 diabetes, %	57.75 (42.49, 73.01)	<0.0001	28.49	29.27
IFG, %	36.37 (26.81, 45.92)	<0.0001	19.92	16.45
IGT, %	8.28 (−1.17, 17.73)	0.0846	−7.25	15.53

#### Association of GRS and lifestyle factors

**Fig. 2:**
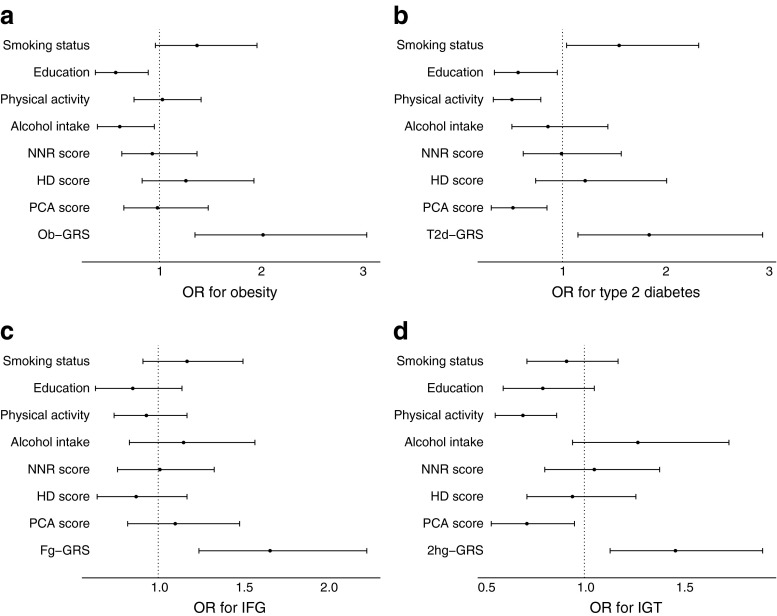
ORs (95% CI) for incidence of (**a**) obesity, (**b**) type 2 diabetes, (**c**) IFG and (**d**) IGT. Smoking status: non-smokers vs current smokers; education: school vs university education; physical activity: inactive vs active; alcohol intake, GRSs and diet scores: 1st vs 4th quartiles

The ob-GRS was independently associated with incidence of obesity (*p* < 0.001, ESM Table 7a) but not significantly associated with weight gain ≥10% (data not shown). Among lifestyle variables, education (*p* < 0.02) and alcohol intake (*p* < 0.03) were significantly inversely associated with incident obesity (Fig. 2a).

### Type 2 diabetes

#### Predictive accuracy

The predictive ability of the genetic model did not significantly differ from that of the lifestyle model (AUC 74% vs 75%; *p*_difference_ = 0.47; Table 2 and Fig. 1b). The predictive ability of these models was lower when the models were not adjusted for age and sex (AUC 68% and 72% for genetic and lifestyle models, respectively). Adding genetic information (65 SNPs) to the lifestyle model significantly improved the predictive ability of the lifestyle model (AUC 80% for the combined model; *p*_difference_ = 0.0003). The sensitivity of the combined model was 40% at a fixed specificity of 90% and the sensitivity was slightly higher in the model that included only lifestyle factors compared with the model that included only genetic information (33% vs 32%).

#### Net reclassification improvement

The cNRI indicated an improvement in prediction of type 2 diabetes after adding genetic information to the lifestyle model (cNRI 58%; *p* < 0.0001; Table 3). The addition of genetic information had a slightly smaller effect on the reclassification of those who developed type 2 diabetes (cNRI 28% for event vs 29% for non-event).

#### Association of GRS and lifestyle factors

The t2d-GRS was significantly associated with the incidence of type 2 diabetes (*p* < 0.02, ESM Table 7b). The PCA score was the only diet score that showed a significant association with incident type 2 diabetes (*p* = 0.01). All other lifestyle risk factors, except alcohol intake, were associated with incident type 2 diabetes: namely, education, smoking and physical activity (all *p* < 0.04, Fig. 2b).

#### Multi-trait genetic information

The analyses including multi-trait genetic information (SNPs associated with type 2 diabetes, fasting glucose, 2 h glucose and obesity) yielded higher predictive accuracies compared with those conducted using only the type 2 diabetes-associated SNPs (Table 4). The genetic model showed a significantly higher predictive accuracy than the lifestyle model (AUC 82% vs 75%, *p*_difference_ = 0.003; ESM Fig. 1). Adding genetic information to lifestyle variables significantly improved the predictive accuracy of the model (AUC 86% for combined model; *p*_difference_ < 0.0001) and resulted in a cNRI of 86% (*p* < 0.0001). The Hosmer–Lemeshow test showed that there was a significant difference between the observed and predicted risk for the genetic (*p* = 0.04) and combined (*p* = 0.006) models. The sensitivities at a fixed specificity of 90% were higher for the genetic and combined models containing multi-trait genetic information compared with the models including only type 2 diabetes-associated SNPs (52% vs 32% for the genetic model and 61% vs 40% for the combined model).

**Table 4:** Predictive ability, model calibration and cNRI for prediction of type 2 diabetes based on genetic factors associated with type 2 diabetes, fasting and 2 h glucose, and obesity alone and in combination with lifestyle factors

Model	AUC (95% CI)	AUC *p* value^a^	Sensitivity (90% specificity)	AIC	Hosmer–Lemeshow *p* value	cNRI (95% CI)^b^	cNRI *p* value
Genetic model	0.815 (0.780, 0.849)	0.003	52%	1304	0.04		
Lifestyle model	0.754 (0.720, 0.789)		33%	1107	0.30		
Combined model	0.856 (0.826, 0.886)	<0.0001	61%	1299	0.006	86.44% (72.07, 100)	<0.0001

^a^AUC *p* values are for genetic and combined models vs lifestyle model

^b^cNRI was calculated by adding the genetic information to lifestyle variables

### IFG and IGT

#### Predictive accuracy

The predictive ability of the genetic model for incident IFG was 66%, 63% for the lifestyle model and 69% for the combined model (Fig. 1c). The difference in predictive ability between the genetic and lifestyle models was not significant (*p*_difference_ = 0.05) but adding genetic information (36 SNPs) to the lifestyle model significantly improved its predictive ability (*p*_difference_ < 0.0001; Table 2).

For incident IGT, the predictive ability of the genetic model (AUC 61%) was significantly lower than that for the lifestyle model (AUC 64%; *p*_difference_ = 0.03; Table 2 and Fig. 1d). Adding the genetic information (nine SNPs) to the lifestyle model significantly improved (*p*_difference_ = 0.03) the predictive ability of the model (AUC 65% for combined model). The sensitivities of all the models were ≥20% at a fixed specificity of 90% for IFG and ≥16% for IGT.

#### Net reclassification improvement

The addition of the genetic information to lifestyle model resulted in a cNRI of 36% (*p* < 0.0001) for IFG but did not result in a significant net reclassification improvement for IGT (cNRI 8%; *p* = 0.08; Table 3).

#### Association of GRS and lifestyle factors

The fg-GRS and 2hg-GRS were significantly associated with incident IFG and incident IGT, respectively (*p* < 0.005; ESM Tables 7c and d). Physical activity (*p* < 0.01) and the PCA diet score (*p* = 0.02) were significantly associated with incident IGT but not with incident IFG (Fig. 2c, d).

## Discussion

This study sought to determine the relative predictive accuracy of genetic and lifestyle variables in incident obesity and type 2 diabetes. We found that, in most scenarios, models testing the predictive accuracy of lifestyle and genetic risk factors yielded comparable results. An exception was for incident IGT, where lifestyle risk factors had greater predictive accuracy than the SNPs associated with 2 h glucose. However, this may reflect the limited information available on the genetics of 2 h glucose, as only nine SNPs were associated with 2 h glucose. Combining genetic and lifestyle information yielded the highest predictive accuracy. For example, in models focused on incident type 2 diabetes, the combination of lifestyle and genetic factors yielded an AUC of 80%, with the lifestyle and genetic models yielding AUCs of 75% and 74%, respectively. The equivalent models for incident obesity yielded AUC values of 79% for the combined model, and 68% and 73% for the lifestyle and genetic models, respectively. The addition of genetic information to the lifestyle model improved the correct classification of participants with type 2 diabetes by 58% and with obesity by 64%.

Genome-wide association studies have made outstanding progress in the identification of loci associated with cardiometabolic traits, increasing interest in the clinical translation of this information. Identification of persons at higher or lower risk for type 2 diabetes and obesity is important to effectively target resources and interventions to reduce disease burden for the patient and for society as a whole [30]. In this sense, genetic information might be useful when seeking to predict later disease in initially healthy people. However, many have highlighted the poor predictive accuracy of models comprised solely of genetic data for obesity and type 2 diabetes [26]. Although it is sometimes assumed lifestyle data are more powerful in this context than genetic data, to our knowledge no previous studies have undertaken a comprehensive head-to-head assessment of established genetic and lifestyle risk markers. Nevertheless, comparisons of clinical risk scores with subsets of the genetic predictors reported here have been described. For example, analyses conducted in the Framingham Offspring Study [6] and in Nordic cohorts [31] showed that 16–18 type 2 diabetes-associated SNPs did not considerably improve the predictive accuracy of clinical risk factors. Analyses focused on 20 type 2 diabetes-associated SNPs in the Whitehall II study reached the same conclusion [5]. However, an extension of the Whitehall II study, which included 65 type 2 diabetes-associated SNPs, showed that the addition of genetic information to the clinical risk factor model significantly improved its predictive accuracy [4]. Previous analysis conducted in the GLACIER cohort showed that adding genetic information (16 fasting glucose and 15 type 2 diabetes-associated loci) to a clinical risk factor model significantly improved the predictive accuracy of incident IFG [32].

The comparison of genetic and lifestyle prediction models is logical because each have independent properties that might prove valuable for the prediction and prevention of type 2 diabetes. Lifestyle modification is expensive but has the potential to substantially reduce diabetes risk in high-risk individuals [33], and thus presents logical intervention targets in those at risk. Genotypes, however, are easily and inexpensively assessed and owing to their salient nature, could in principle be used for risk prediction from the very earliest stages of life. Importantly, quantitative disease phenotypes, such as blood glucose or cholesterol, which are often used in clinical risk scores, are outcomes of unhealthful lifestyles and unfavourable genetic profiles; thus, these phenotypes are typically part of the pathophysiology of disease rather than primordial risk factors. Thus, comparing genetic and clinical prediction scores is less logical in some respects.

An important limitation of the current study is that lifestyle factors were assessed using questionnaires, which are prone to error and response bias, undermining the accuracy with which such data can be used to predict disease. However, even in clinical practice, lifestyle is usually assessed with questions, meaning that these are limitations that one will often face when using lifestyle data to predict disease, regardless of the setting. Another important consideration is that the loci were selected from existing genetic association studies of cross-sectional data. While these associations are statistically robust, it is likely that hitherto unknown loci exist that predict changes in quantitative measures of glucose and body composition, and that some of the loci included here are uninformative because they play no role in trait change. These factors, to some extent, likely undermine the predictive ability of the genetic models. Thus, as progress is made in both lifestyle and genetic epidemiology, it is likely that the predictive accuracy of models will improve. Finally, we did not model interactions between lifestyle and genetic variables, as our study is underpowered for this task. However, a previous study concluded that the inclusion of gene–gene and gene–environment interaction data into type 2 diabetes risk prediction models is unlikely to dramatically improve the sensitivity or specificity of the models [34].

In conclusion, both lifestyle and genetic information yield reasonably high predictive accuracies for type 2 diabetes and obesity incidence in the GLACIER Study, and neither outperformed the other. Adding genetic information to models containing lifestyle variables significantly improved the prediction of incident type 2 diabetes and obesity. These findings imply that genetic profiling might help the identification of persons who are susceptible to developing obesity and type 2 diabetes in the future, and that the combination of both genetic and lifestyle information offers high predictive accuracy for those diseases.

## Electronic supplementary material

ESM Fig. 1 (PDF 97 kb)

ESM Table 1 (PDF 128 kb)

ESM Table 2 (PDF 134 kb)

ESM Table 3 (PDF 88 kb)

ESM Table 4 (PDF 59 kb)

ESM Table 5 (PDF 66 kb)

ESM Table 6 (PDF 82 kb)

ESM Table 7 (PDF 114 kb)

## Abbreviations

2hg-GRS: 2 h glucose genetic risk score
AIC: Akaike’s information criterion
cNRI: Continuous net reclassification improvement
FFQ: Food frequency questionnaire
fg-GRS: Fasting glucose genetic risk score
GLACIER: Gene–Lifestyle Interactions and Complex Traits Involved in Elevated Disease Risk
GRS: Genetic risk score
HD: Healthy diet
IFG: Impaired fasting glucose
IGT: Impaired glucose tolerance
LD: Linkage disequilibrium
NNR: Nordic nutrition recommendation
ob-GRS: Obesity genetic risk score
PCA: Principal component analysis
SNP: Single nucleotide polymorphism
t2d-GRS: Type 2 diabetes genetic risk score
VHU: Västerbotten Health Survey
