# Supplementary material for: Innate biology versus lifestyle behaviour in the aetiology of obesity and type 2 diabetes: the GLACIER Study
Source: Diabetologia. 2015 Dec 1;59:462–71. doi: 10.1007/s00125-015-3818-y (PMC4742501; doi:10.1007/s00125-015-3818-y)
Supplement: Supplementary file 1 — (PDF 97 kb) [file 125_2015_3818_MOESM1_ESM.pdf]

ESM Figure 1. AUCs for type 2 diabetes incidence using a combination of SNPs associated with obesity, fasting glucose, 2h glucose and type 2 diabetes.

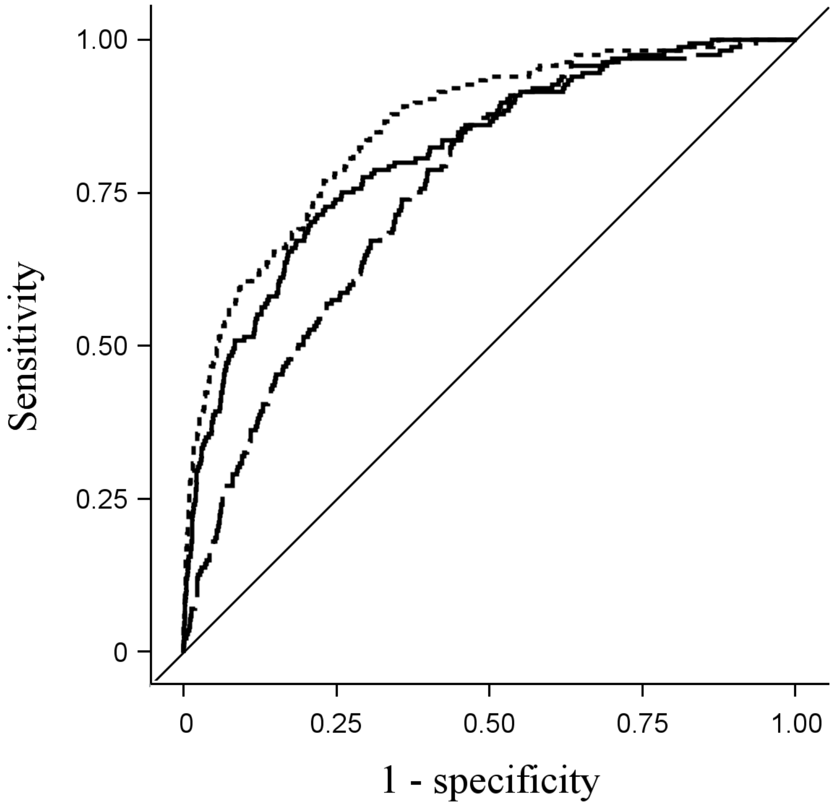

Solid line, genetic model; dashed line, lifestyle model; dotted line, combined model
