# Supplementary material for: Innate biology versus lifestyle behaviour in the aetiology of obesity and type 2 diabetes: the GLACIER Study
Source: Diabetologia. 2015 Dec 1;59:462–71. doi: 10.1007/s00125-015-3818-y (PMC4742501; doi:10.1007/s00125-015-3818-y)
Supplement: Supplementary file 2 — (PDF 128 kb) [file 125_2015_3818_MOESM2_ESM.pdf]

ESM Table 1 Quality control of 97 BMI associated genetic variants (1) in the GLACIER Study (n= 5,726)

Original loci	Nearest Gene	Proxy used	R^2 (D')	Chr:position	Effect allele /Other	Original EAF	GLACIER EAF	HWE *p*-value
rs17024393	*GNAT2*			1:109,956,211	C/T	0.04	0.03	0.01
rs543874	*SEC16B*			1:176,156,103	G/A	0.19	0.22	0.98
rs2820292	*NAV1*			1:200,050,910	C/A	0.56	0.62	0.18
rs977747	*TAL1*			1:47,457,264	T/G	0.40	0.37	0.84
rs657452	*AGBL4*			1:49,362,434	A/G	0.39	0.40	0.07
rs11583200	*ELAVL4*			1:50,332,407	C/T	0.40	0.41	0.0005
rs3101336	*NEGR1*			1:72,523,773	C/T	0.61	0.58	0.49
rs12566985	*FPGT-TNNI3K*	rs1514175	0.97 (1.0)	1:74,764,232	A/G	0.45	0.43	0.60
rs12401738	*FUBP1*			1:78,219,349	A/G	0.35	0.37	0.04
rs11165643	*PTBP2*			1:96,696,685	T/C	0.58	0.57	0.03

rs2121279	*LRP1B*			2:142,759,755	T/C	0.15	0.12	0.64
rs1460676	*FIGN*			2:164,275,935	C/T	0.18	0.13	0.08
rs1528435	*UBE2E3*			2:181,259,207	T/C	0.63	0.63	0.33
rs17203016	*CREB1*			2:207,963,763	G/A	0.19	0.19	0.87
rs7599312	*ERBB4*			2:213,121,476	G/A	0.72	0.71	0.09
rs492400	*USP37*			2:219,057,996	C/T	0.42	0.42	0.08
rs2176040	*LOC646736*	rs2972143	0.96 (1.0)	2:226,824,609	A/G	0.37	0.41	0.48
rs10182181	*ADCY3*			2:25,003,800	G/A	0.46	0.44	0.28
rs11126666	*KCNK3*			2:26,782,315	A/G	0.28	0.29	0.20
rs1016287	*FLJ30838*			2:59,159,129	T/C	0.29	0.27	0.91
rs11688816	*EHBP1*			2:62,906,552	G/A	0.52	0.53	0.33
rs13021737	*TMEM18*			2:622,348	G/A	0.83	0.82	0.70
rs6091540	*ZFP64*			20:50,521,269	C/T	0.72	0.72	0.35
rs2836754	*ETS2*			21:39,213,610	C/T	0.60	0.67	0.40
rs16851483	*RASA2*	rs2035935	0.95 (1.0)	3:142,788,703	G/A	0.07	0.06	0.65

rs1516725	*ETV5*	rs4234589	1.0 (1.0)	3:187,301,576	A/G	0.87	0.87	0.33
rs6804842	*RARB*			3:25,081,441	G/A	0.57	0.60	0.73
rs2365389	*FHIT*			3:61,211,502	C/T	0.58	0.59	0.05
rs3849570	*GBE1*			3:81,874,802	A/C	0.36	0.35	0.44
rs13078960	*CADM2*	rs7622475	0.95 (1.0)	3:85,912,107	C/T	0.20	0.13	0.04
rs13107325	*SLC39A8*			4:103,407,732	T/C	0.07	0.03	0.73
rs11727676	*HHIP*			4:145,878,514	T/C	0.91	0.93	0.70
rs10938397	*GNPDA2*			4:44,877,284	G/A	0.43	0.38	0.98
rs17001654	*SCARB2*	rs17001561	1.0 (1.0)	4:77,315,142	A/G	0.15	0.13	0.19
rs7715256	*GALNT10*			5:153,518,086	G/T	0.42	0.37	0.54
rs2112347	*POC5*			5:75,050,998	T/G	0.63	0.68	0.15
rs9400239	*FOXO3*			6:109,084,356	C/T	0.69	0.72	0.02
rs9374842	*LOC285762*			6:120,227,364	T/C	0.74	0.74	0.98
rs13201877	*IFNGR1*			6:137,717,234	G/A	0.14	0.15	0.03
rs13191362	*PARK2*			6:162,953,340	A/G	0.88	0.84	0.37

rs205262	*C6orf106*			6:34,671,142	G/A	0.27	0.24	0.51
rs2033529	*TDRG1*			6:40,456,631	G/A	0.29	0.27	0.06
rs2207139	*TFAP2B*	rs734597	0.90 (1.0)	6:50,944,238	A/G	0.18	0.20	0.95
rs1167827	*HIP1*			7:75,001,105	G/A	0.55	0.58	0.43
rs2245368	*PMS2L11*			7:76,446,079	C/T	0.18	0.20	0.29
rs9641123	*CALCR*	rs5014937	0.67 (0.96)	7:93,017,926	C/A	0.43	0.43	0.16
rs6465468	*ASB4*			7:95,007,450	T/G	0.31	0.26	0.63
rs17405819	*HNF4G*			8:76,969,139	T/C	0.70	0.72	0.61
rs16907751	*ZBTB10*			8:81,538,012	C/T	0.91	0.87	0.49
rs2033732	*RALYL*			8:85,242,264	C/T	0.75	0.76	0.49
rs6477694	*EPB41L4B*			9:110,972,163	C/T	0.37	0.39	0.02
rs1928295	*TLR4*			9:119,418,304	T/C	0.55	0.51	0.63
rs10733682	*LMX1B*			9:128,500,735	A/G	0.48	0.47	0.62
rs4740619	*C9orf93*			9:15,624,326	T/C	0.54	0.55	0.74
rs10968576	*LINGO2*			9:28,404,339	G/A	0.32	0.34	0.12

rs17094222	*HIF1AN*			10:102,385,430	C/T	0.21	0.21	0.25
rs11191560	*NT5C2*			10:104,859,028	C/T	0.09	0.08	0.09
rs7903146	*TCF7L2*			10:114,748,339	C/T	0.71	0.79	0.87
rs7899106	*GRID1*			10:87,400,884	G/A	0.05	0.04	0.65
rs12286929	*CADM1*			11:114,527,614	G/A	0.52	0.52	0.35
rs11030104	*BDNF*	rs7103411	0.95 (1.0)	11:27,656,701	T/C	0.79	0.78	0.09
rs2176598	*HSD17B12*			11:43,820,854	T/C	0.25	0.22	0.74
rs3817334	*MTCH2*			11:47,607,569	T/C	0.41	0.43	0.94
rs4256980	*TRIM66*	rs7113874	1.0 (1.0)	11:8,644,592	C/T	0.65	0.64	0.81
rs11057405	*CLIP1*			12:121,347,850	G/A	0.90	0.89	0.67
rs7138803	*BCDIN3D*			12:48,533,735	A/G	0.38	0.45	0.75
rs12016871	*MTIF3*	rs1885988	0.82 (0.95)	13:26,908,262	C/T	0.20	0.25	0.79
rs12429545	*OLFM4*			13:53,000,207	A/G	0.13	0.16	0.95
rs9540493	*MIR548X2*			13:65,103,705	A/G	0.45	0.47	0.98
rs1441264	*MIR548A2*			13:78,478,920	A/G	0.61	0.65	0.16

rs10132280	*STXBP6*			14:24,998,019	C/A	0.68	0.72	0.42
rs12885454	*PRKD1*			14:28,806,589	C/A	0.64	0.64	0.99
rs11847697	*PRKD1*	rs10134820	0.74 (1.0)	14:29,571,636	T/C	0.04	0.03	0.36
rs7141420	*NRXN3*			14:78,969,207	T/C	0.53	0.52	0.73
rs3736485	*DMXL2*			15:49,535,902	A/G	0.45	0.43	0.55
rs16951275	*MAP2K5*	rs2241420	1.0 (1.0)	15:65,869,870	G/A	0.78	0.83	0.93
rs7164727	*LOC100287559*			15:70,881,044	T/C	0.67	0.70	0.28
rs12446632	*GPRC5B*			16:19,842,890	G/A	0.87	0.90	0.86
rs2650492	*SBK1*			16:28,240,912	A/G	0.30	0.32	0.49
rs3888190	*ATP2A1*			16:28,796,987	A/C	0.40	0.41	0.58
rs4787491	*INO80E*			16:29,922,838	G/A	0.51	0.48	0.51
rs758747	*NLRC3*			16:3,567,359	T/C	0.27	0.26	0.84
rs9925964	*KAT8*			16:31,037,396	A/G	0.62	0.63	0.89
rs2080454	*CBLN1*			16:47,620,091	C/A	0.41	0.44	0.28
rs1558902	*FTO*	rs1421085	1.0 (1.0)	16:52,358,455	C/T	0.42	0.42	0.005

rs9914578	*SMG6*	17:1,951,886	G/C	0.23	0.24	0.77
rs1000940	*RABEP1*	17:5,223,976	G/A	0.32	0.34	0.90
rs12940622	*RPTOR*	17:76,230,166	G/A	0.57	0.57	0.28
rs1808579	*C18orf8*	18:19,358,886	C/T	0.53	0.55	0.55
rs7239883	*LOC284260*	18:38,401,669	G/A	0.39	0.38	0.30
rs7243357	*GRP*	18:55,034,299	T/G	0.81	0.81	0.62
rs6567160	*MC4R*	18:55,980,115	C/T	0.24	0.26	0.03
rs17724992	*PGPEP1*	19:18,315,825	A/G	0.75	0.77	0.56
rs29941	*KCTD15*	19:39,001,372	G/A	0.67	0.63	0.84
rs2075650	*TOMM40*	19:50,087,459	A/G	0.85	0.85	0.04
rs2287019	*QPCTL*	19:50,894,012	C/T	0.80	0.83	0.63
rs3810291	*ZC3H4*	19:52,260,843	A/G	0.67	0.69	0.24

Chr: Chromosome. EAF: Effect allele frequency. HWE: Hardy-Weinberg equilibrium
