# Supplementary material for: Innate biology versus lifestyle behaviour in the aetiology of obesity and type 2 diabetes: the GLACIER Study
Source: Diabetologia. 2015 Dec 1;59:462–71. doi: 10.1007/s00125-015-3818-y (PMC4742501; doi:10.1007/s00125-015-3818-y)
Supplement: Supplementary file 3 — (PDF 134 kb) [file 125_2015_3818_MOESM3_ESM.pdf]

ESM Table 2 Quality control of 36 fasting glucose, 9 2h glucose (3) and 65 type 2 diabetes-associated genetic variants (2) in the GLACIER Study (n= 5,726).

Loci	Nearest Gene	Proxy used	R² (D')	Chr: position	Effect allele/Other	Original EAF	GLACIER EAF	HWE *p*-value
**Fasting glucose**								
rs340874	*PROX1*			1:212,225,879	C/T	0.52	0.53	0.50
rs780094	*GCKR*			2:27,594,741	C/T	0.61	0.71	0.77
rs560887	*G6PC2*			2:169,471,394	C/T	0.70	0.71	0.04
rs11715915	*AMT*			3:49,430,334	C/T	0.68	0.66	0.13
rs11708067	*ADCY5*			3:124,548,468	A/G	0.79	0.79	0.40
rs1280	*SLC2A2*			3:172,195,984	T/C	0.86	0.86	0.58
rs7651090	*IGF2BP2*			3:186,996,086	G/A	0.31	0.26	0.26
rs7708285	*ZBED3*			5:76,461,623	G/A	0.27	0.23	0.54
rs4869272	*PCSK1/MIR583*			5:95,565,204	T/C	0.69	0.65	0.33

rs17762454	*RREB1*	6:7,158,199	T/C	0.26	0.21	0.66
rs9368222	*CDKAL1*	6:20,794,975	A/C	0.28	0.24	0.43
rs2191349	*DGKB/TMEM195*	7:15,030,834	T/G	0.53	0.51	0.06
rs2908289	*GCK*	7:44,190,467	A/G	0.16	0.15	0.02
rs6943153	*GRB10*	7:50,759,073	T/C	0.34	0.33	0.13
rs983309	*PPP1R3B/LOC157273*	8:9,215,142	T/G	0.12	0.16	0.46
rs11558471	*SLC30A8*	8:118,254,914	A/G	0.68	0.69	0.05
rs10814916	*GLIS3*	9:4,283,150	C/A	0.51	0.48	0.93
rs10811661	*CDKN2B*	9:22,124,094	T/C	0.82	0.77	0.08
rs16913693	*IKBKAP*	9:110,720,180	T/G	0.97	0.97	0.37
rs3829109	*DNLZ*	9:138,376,587	G/A	0.71	0.70	0.86
rs11195502	*ADRA2A*	10:113,029,657	C/T	0.91	0.93	0.48
rs7903146	*TCF7L2*	10:114,748,339	T/C	0.28	0.21	0.87
rs11607883	*CRY2*	11:45,796,285	G/A	0.48	0.50	0.92
rs11039182	*MADD*	11:47,303,299	T/C	0.73	0.77	0.33

rs174576	*FADS1*	11:61,360,086	C/A	0.65	0.66	0.06
rs11603334	*ARAP1*	11:72,110,633	G/A	0.83	0.81	0.06
rs10830963	*MTNR1B*	11:92,348,358	G/C	0.29	0.28	0.28
rs2657879	*GLS2*	12:55,151,605	G/A	0.18	0.15	0.71
rs10747083	*P2RX2*	12:13,551,691	A/G	0.66	0.73	0.71
rs11619319	*PDX1*	13:27,385,599	G/A	0.23	0.20	0.10
rs576674	*KL*	13:32,452,302	G/A	0.15	0.17	0.74
rs3783347	*WARS*	14:99,909,014	G/T	0.79	0.78	0.18
rs4502156	*VPS13C/C2CD4A/B*	15:60,170,447	T/C	0.55	0.52	0.94
rs2302593	*GIPR*	19:50,888,474	C/G	0.50	0.50	0.81
rs6113722	*FOXA2*	20:22,505,099	G/A	0.96	0.98	0.44
rs6072275	*TOP1*	20:39,177,319	A/G	0.16	0.19	0.64
**2h glucose**						
rs1260326	*GCKR*	2:27,584,444	T/C	0.38	0.30	0.38
rs11717195	*ADCY5*	3:124,565,088	T/C	0.79	0.79	0.43

rs12255372	*TCF7L2*			10:114,798,892	T/G	0.24	0.20	0.96
rs1436958	*VPS13C/C2CD4A/B*			15:60,126,089	T/G	0.50	0.49	0.06
rs11672660	*GIPR*			19:50,872,024	T/C	0.22	0.19	0.63
rs6975024	*GCK*			7:44,198,411	C/T	0.15	0.15	0.01
rs11782386	*PPP1R3B*			8:9,239,197	C/T	0.87	0.88	0.76
rs1019503	*ERAP2*			5:96,280,573	A/G	0.48	0.47	0.05
rs7651090	*IGF2BP2*			3:186,996,086	G/A	0.30	0.26	0.26
**Type 2 Diabetes**								
rs10923931	*NOTCH2*	rs7515431	1.0 (1.0)	1:120,315,071	T/C	0.12	0.09	0.71
rs2075423	*PROX1*	rs340874	0.49 (1.0)	1:212,225,879	C/T	0.62	0.53	0.50
rs780094	*GCKR*			2:27,594,741	C/T	0.61	0.71	0.77
rs10203174	*THADA*			2:43,543,534	C/T	0.89	0.92	0.14
rs243088	*BCL11A*	rs243083	1.0 (1.0)	2:60,427,374	G/A	0.45	0.40	0.15
rs7569522	*RBMS1*			2:161,054,693	A/G	0.44	0.47	0.98
rs13389219	*GRB14*	rs1128249	1.0 (1.0)	2:165,236,870	G/T	0.60	0.58	0.99

rs2943640	*IRS1*			2:226,801,829	C/A	0.63	0.59	0.68
rs1801282	*PPARG*	rs2197423	1.0 (1.0)	3:12,366,583	G/A	0.86	0.85	0.06
rs1496653	*UBE2E2*			3:23,429,794	A/G	0.75	0.73	0.88
rs12497268	*PSMD6*			3:64,065,403	G/C	0.80	0.81	0.53
rs6795735	*ADAMTS9*			3:64,680,405	C/T	0.59	0.64	0.88
rs11717195	*ADCY5*			3:124,565,088	T/C	0.77	0.79	0.43
rs4402960	*IGF2BP2*			3:186,994,381	T/G	0.33	0.26	0.22
rs17301514	*ST64GAL1*			3:188,096,103	A/G	0.13	0.12	0.34
rs6819243	*MAEA*			4:1,283,245	T/C	0.96	0.97	0.95
rs4458523	*WFS1*	rs10012946	1.0 (1.0)	4:6,344,251	C/T	0.57	0.57	0.49
rs459193	*ANKRD55*			5:55,842,508	G/A	0.70	0.75	0.50
rs6878122	*ZBED3*			5:76,463,067	G/A	0.28	0.25	0.30
rs7756992	*CDKAL1*			6:20,787,688	G/A	0.29	0.24	0.21
rs4299828	*ZFAND3*			6:38,285,645	A/G	0.79	0.86	0.61
rs3734621	*KCNK16*			6:39,412,189	C/A	0.03	0.01	0.01

rs17168486	*DGKB*			7:14,864,807	T/C	0.19	0.18	1.00
rs849135	*JAZF1*			7:28,162,938	G/A	0.52	0.55	0.80
rs10278336	*GCK*	rs3824065	1.0 (1.0)	7:44,213,783	C/T	0.50	0.51	0.11
rs17867832	*GCC1*	rs10229583	0.14 (0.58)	7:127,034,139	G/A	0.91	0.71	0.59
rs13233731	*KLF14*			7:130,088,229	G/A	0.51	0.48	0.44
rs516946	*ANK1*			8:41,638,405	C/T	0.76	0.80	0.35
rs7845219	*TP53INP1*			8:96,006,678	T/C	0.52	0.57	0.65
rs3802177	*SLC30A8*			8:118,254,206	G/A	0.66	0.70	0.08
rs10758593	*GLIS3*			9:4,282,083	A/G	0.42	0.37	0.53
rs16927668	*PTPRD*			9:8,359,533	T/C	0.24	0.21	0.60
rs10811661	*CDKN2A/B*			9:22,124,094	T/C	0.82	0.77	0.08
rs17791513	*TLE4*			9:81,095,410	A/G	0.91	0.89	0.77
rs2796441	*TLE1*			9:83,498,768	G/A	0.57	0.58	0.78
rs11257655	*CDC123/CAMK1D*			10:12,347,900	T/C	0.23	0.22	0.96
rs12242953	*VPS26A*			10:70,535,348	G/A	0.93	0.96	0.03

rs12571751	*ZMIZ1*			10:80,612,637	A/G	0.52	0.57	0.65
rs1111875	*HHEX/IDE*			10:94,452,862	C/T	0.58	0.52	0.36
rs7903146	*TCF7L2*			10:114,748,339	T/C	0.27	0.21	0.87
rs2334499	*DUSP8*			11:1,653,425	T/C	0.43	0.44	0.48
rs163184	*KCNQ1*			11:2,803,645	G/T	0.50	0.51	0.02
rs5215	*KCNJ11*			11:1,7365,206	C/T	0.41	0.42	0.23
rs1552224	*ARAP1 (CENTD2)*			11:72,110,746	A/C	0.81	0.81	0.08
rs10830963	*MTNR1B*			11:92,348,358	G/C	0.31	0.28	0.28
rs11063069	*CCND2*			12:4,244,634	G/A	0.21	0.25	0.56
rs10842994	*KLHDC5*			12:27,856,417	C/T	0.80	0.80	0.62
rs2261181	*HMGA2*			12:64,498,585	T/C	0.10	0.09	0.59
rs7955901	*TSPAN8/LGR5*			12:69,719,560	C/T	0.45	0.44	0.52
rs12427353	*HNF1A (TCF1)*	rs7965349	0.77 (0.93)	12:119,956,314	C/T	0.79	0.81	0.13
rs1359790	*SPRY2*			13:79,615,157	G/A	0.72	0.75	0.14
rs4502156	*C2CD4A*			15:60,170,447	T/C	0.52	0.52	0.94

rs7177055	*HMG20A*			15:75,619,817	A/G	0.68	0.72	0.77
rs11634397	*ZFAND6*			15:78,219,277	G/A	0.64	0.61	0.80
rs2007084	*AP3S2*			15:88,146,339	G/A	0.92	0.91	0.48
rs12899811	*PRC1*			15:89,345,080	G/A	0.31	0.31	0.003
rs9936385	*FTO*	rs9923233	0.935(1.000)	16:52,376,699	C/G	0.41	0.41	0.009
rs7202877	*BCAR1*			16:73,804,746	T/G	0.89	0.90	1.0
rs2447090	*SRR*			17:2,245,724	A/G	0.62	0.66	0.15
rs11651052	*HNF1B*	rs11651755	1.000(1.000)	17:33,173,953	C/T	-	0.43	0.13
rs12970134	*MC4R*	rs11663816	1.000(1.000)	18:56,027,207	C/T	0.27	0.29	0.05
rs10401969	*CILP2*			19:19,268,718	C/T	0.08	0.08	0.04
rs8182584	*PEPD*			19:38,601,550	T/G	0.38	0.40	0.36
rs8108269	*GIPR*			19:50,850,353	G/T	0.31	0.30	0.42
rs4812829	*HNF4A*			20:42,422,681	A/G	0.19	0.20	0.88

Chr: Chromosome. EAF: Effect allele frequency. HWE: Hardy-Weinberg equilibrium
