# Supplementary material for: Innate biology versus lifestyle behaviour in the aetiology of obesity and type 2 diabetes: the GLACIER Study
Source: Diabetologia. 2015 Dec 1;59:462–71. doi: 10.1007/s00125-015-3818-y (PMC4742501; doi:10.1007/s00125-015-3818-y)
Supplement: Supplementary file 4 — (PDF 88 kb) [file 125_2015_3818_MOESM4_ESM.pdf]

ESM Table 3 Diet criteria according to the Nordic Nutrition Recommendations (20)

	Men			Women		
**Diet variables intake**	**Recommendation**	**Upper limit**	**N following guidelines**	**Recommendation**	**Upper limit**	**N following guidelines**
Carbohydrate (%E/day)	45-60		820 (65.2)	45-60		1629 (79.9)
Sucrose (%E/day)	<10		1049 (83.4)	<10		1743 (85.5)
Protein (%E/day)	10-20		1227 (97.5)	10-20		2005 (98.3)
Total fat (%E/day)	25-40		908 (72.2)	25-40		1671 (82.0)
Saturated fat (%E/day)	<10		52 (4.1)	<10		151 (7.4)
EFA (%E/day)	>3		1218 (96.8)	>3		1897 (93.0)
MUFA (%E/day)	10-20		1105 (87.8)	10-20		1282 (62.9)
PUFA (%E/day)	5-10		624 (49.6)	5-10		639 (31.3)
Fiber (g/day)	≥25		327 (26.0)	≥25		365 (17.9)
Salt (g/day)	<6		1317 (99.6)	<6		2122 (100)
Vitamin A (μg/day)	900	3000	605 (48.1)	700	3000	851 (41.7)

Vitamin D (μg/day)	10	100	145 (11.5)	10	100	11 (0.5)
Vitamin E (mg/day)	10	300	230 (18.3)	8	300	340 (16.7)
Thiamin (mg/day)	1.3		704 (56.0)	1.1		1190 (58.4)
Riboflavin (mg/day)	1.5		690 (54.9)	1.2		1240 (60.8)
Niacin (mg/day)	18	900	537 (42.7)	14	900	892 (43.8)
Vitamin B6 (mg/day)	1.5	25	1068 (84.9)	1.2	25	1835 (90.0)
Folate (μg/day)	300	1000	327 (26.0)	300	1000	384 (18.8)
Vitamin B12 (μg/day)	2		1223 (97.2)	2		1886 (92.5)
Vitamin C (mg/day)	75	1000	606 (48.2)	75	1000	1151 (56.5)
Calcium (mg/day)	800	2500	742 (59.0)	800	2500	958 (47.0)
Phosphorus (mg/day)	600	3000	1230 (97.8)	600	3000	1955 (95.9)
Potassium (g/day)	3.5	3.7	78 (6.2)	3.1	3.7	507 (24.9)
Magnesium (mg/day)	350		556 (44.2)	280		1005 (49.3)
Iron (mg/day)	9	25	1053 (83.7)	9	25	1601 (78.5)
Zinc (mg/day)	9	25	637 (50.6)	7	25	1230 (60.3)

Iodine (µg/day)	150	600	418 (33.2)	150	600	273 (13.4)
Selenium (µg/day)	60	300	9 (0.7)	50	300	8 (0.4)

Data are expressed as n (%). %E/day: % of Energy per day
