# Supplementary material for: Innate biology versus lifestyle behaviour in the aetiology of obesity and type 2 diabetes: the GLACIER Study
Source: Diabetologia. 2015 Dec 1;59:462–71. doi: 10.1007/s00125-015-3818-y (PMC4742501; doi:10.1007/s00125-015-3818-y)
Supplement: Supplementary file 5 — (PDF 59 kb) [file 125_2015_3818_MOESM5_ESM.pdf]

ESM Table 4 Summary of the results of the Principal Component Analysis conducted on macronutrients (n= 3,297)

**Macronutrients intake**	**Factor loading**
Carbohydrates	-0.93
Protein	-0.12
Total fat	0.97
Saturated fat	0.81
Monounsaturated fatty acids	0.84
Polyunsaturated fatty acids	0.64
Essential fatty acids	0.53
Fiber	-0.66
Eigenvalue	4.30
% of total variance	54%
KMO (Kaiser-Meyer-Olkin)	0.71
