# Supplementary material for: Innate biology versus lifestyle behaviour in the aetiology of obesity and type 2 diabetes: the GLACIER Study
Source: Diabetologia. 2015 Dec 1;59:462–71. doi: 10.1007/s00125-015-3818-y (PMC4742501; doi:10.1007/s00125-015-3818-y)
Supplement: Supplementary file 6 — (PDF 66 kb) [file 125_2015_3818_MOESM6_ESM.pdf]

ESM Table 5 Basic descriptive statistics of lifestyle variables, diet and genetic scores at baseline in the GLACIER Study

Variables	Men (n= 1,322)	Women (n= 2,122)	All (n= 3,444)
Smoking (%: current/ex/non)	20.2/26.3/53.5	23.5/19.1/57.4	22.2/21.9/55.9
Education (%: school/high-school/university)	28.9/52.5/18.5	26.9/47.4/25.7	27.7/49.4/22.9
Physical activity (%: inactive/active)	72.9/27.1	69.8/30.2	71.0/29.0
Total energy intake (kcal/day)	2,114±644	1,642±483	1,822±596
Carbohydrate intake (g/day)	251.9±81.8	208.1±67.6	224.8±76.4
Sucrose intake (g/day)	38.5±20.8	30.3±15.9	33.4±18.4
Protein intake (g/day)	74.1±24.6	59.7±18.1	65.2±22.0
Total fat intake (g/day)	85.0±31.0	59.9±21.1	69.5±28.1
Saturated fat intake (g/day)	36.1±14.3	25.5±9.6	29.5±12.7
Essential fatty acids intake (g/day) [a]	11.2±5.0	7.9±3.6	9.15±4.5
MUFA intake (g/day)	29.8±10.9	19.4±6.6	23.4±9.9
PUFA intake (g/day)	12.6±5.6	8.7±3.8	10.2±4.9
Fiber intake (g/day)	20.6±7.9	19.0±6.9	19.6±7.3
Alcohol intake (g/day)	5.7±5.3	2.3±2.7	3.6±4.2
Salt intake (g/day)	2.6±0.9	1.9±0.6	2.2±0.8
Vitamin A intake (mg/day)	0.98±0.47	0.71±0.36	0.81±0.42
Vitamin D intake (μg/day)	6.8÷2.6	4.6±1.7	5.4±2.3
Vitamin E intake (mg/day)	7.6±2.9	6.1±2.2	6.7±2.6
Thiamin intake (mg/day)	1.4±0.5	1.2±0.4	1.3±0.5

Riboflavin intake (mg/day)	1.7±0.6	1.4±0.5	1.5±0.6
Niacin intake (mg/day)	17.9±6.1	14.0±4.4	15.5±5.5
Vitamin B6 intake (mg/day)	2.3±0.8	1.9±0.7	2.1±0.7
Folate intake (µg/day)	252.7±90.0	236.7±91.7	242.8±91.4
Vitamin B12 intake (µg/day)	5.5±2.7	4.1±1.8	4.6±2.3
Vitamin C intake (mg/day)	83.2±48.5	92.3±52.0	88.8±50.9
Calcium intake (mg/day)	937.0±402.0	812.5±320.2	860.0±358.7
Phosphorus intake (mg/day)	1,432±480	1,116±349	1,237±432
Potassium intake (mg/day)	3,745±1227	3,333±1050	3,490±1139
Magnesium intake (mg/day)	348.3±107.4	288±84.4	310.8±98.4
Iron intake (mg/day)	15.6±5.7	12.7±4.4	13.8±5.1
Zinc intake (mg/day)	9.4±3.2	7.9±2.6	8.5±2.9
Iodine intake (µg/day)	135.3±63.0	104.6±43.3	116.3±53.8
Selenium intake (µg/day)	25.4±9.7	20.9±6.9	22.6±8.4
NNR score	15.5±4.6	15.1±4.3	15.2±4.4
HD score	10.1±3.7	13.3±3.9	12.0±4.1
ob-GRS (effect allele)	91.6±6.4	91.6±6.3	91.6±6.3
t2d-GRS (effect allele)	68.7±5.1	68.7±4.9	68.7±5.0
fg-GRS (effect allele)	37.9±3.6	38.1±3.5	38.0±3.5
2hg-GRS (effect allele)	7.5±1.8	7.5±1.8	7.5±1.8

Data are expressed as mean±SD for quantitative variables and as % for qualitative variables

[a]Intake of omega 3 and omega 6 fatty acids
