# Supplementary material for: Innate biology versus lifestyle behaviour in the aetiology of obesity and type 2 diabetes: the GLACIER Study
Source: Diabetologia. 2015 Dec 1;59:462–71. doi: 10.1007/s00125-015-3818-y (PMC4742501; doi:10.1007/s00125-015-3818-y)
Supplement: Supplementary file 7 — (PDF 82 kb) [file 125_2015_3818_MOESM7_ESM.pdf]

ESM Table 6 Predictive ability, model calibration and cNRI for prediction of weight gain ≥ 10% based on lifestyle and genetic factors alone and in combination.

Model	AUC (95% CI)	AUC *p* value[a]	Sensitivity (90% specificity)	AIC	Hosmer-Lemeshow *p*-value	cNRI (95% CI)[b]	cNRI *p* value
Genetic model	0.650 (0.626, 0.674)	0.87	23%	3206	0.92		
Lifestyle model	0.648 (0.625, 0.672)		23%	3096	0.26		
Combined model	0.675 (0.652, 0.698)	0.0004	26%	3222	0.71	25.93% (17.29, 34.58)	<0.0001

[a]AUC *p* values are for genetic and combined models vs lifestyle model. [b]cNRI was calculated by adding the genetic information to lifestyle variables
