# Supplementary material for: Innate biology versus lifestyle behaviour in the aetiology of obesity and type 2 diabetes: the GLACIER Study
Source: Diabetologia. 2015 Dec 1;59:462–71. doi: 10.1007/s00125-015-3818-y (PMC4742501; doi:10.1007/s00125-015-3818-y)
Supplement: Supplementary file 8 — (PDF 114 kb) [file 125_2015_3818_MOESM8_ESM.pdf]

ESM Table 7 ORs for prediction of incident (**a**) obesity, (**b**) type 2 diabetes, (**c**) IFG and (**d**) IGT according to lifestyle risk factors and GRSs.

a) Obesity

	Regression model (OR, 95% CI)								
	**NNR Score**			**HD Score**			**PCA Score**		
**Variables**	**Genetic** (n=1,511)	**Lifestyle** (n= 1,511)	**Combined** (n= 1,511)	**Genetic** (n=1557)	**Lifestyle** (n= 1,557)	**Combined** (n= 1,557)	**Genetic** (n= 1,511)	**Lifestyle** (n= 1,511)	**Combined** (n= 1,511)
Smoking status	-	1.36 (0.95-1.93)	1.37 (0.96-1.96)	-	1.34 (0.94-1.90)	1.35 (0.95-1.92)	-	1.36 (0.96-1.94)	1.38 (0.97-1.96)
Education	-	**0.58** (0.37-0.89)	**0.58** (0.37-0.90)	-	**0.57** (0.37-0.89)	**0.57** (0.37-0.89)	-	**0.57** (0.37-0.89)	**0.57** (0.37-0.89)
Alcohol intake	-	**0.62** (0.40-0.96)	**0.61** (0.39-0.95)	-	**0.61** (0.40-0.94)	**0.61** (0.39-0.94)	-	**0.62** (0.40-0.96)	**0.61** (0.39-0.95)
Physical activity	-	1.05 (0.77-1.44)	1.03 (0.75-1.41)	-	1.03 (0.76-1.41)	1.02 (0.75-1.39)	-	1.05 (0.77-1.43)	1.03 (0.75-1.41)
Ob-GRS	**2.02** (1.36-3.02)	-	**2.03** (1.35-3.05)	**1.97** (1.33-2.93)	-	**1.97** (1.32-2.94)	**2.02** (1.36-3.02)	-	**2.02** (1.35-3.04)
NNR Score	-	0.91 (0.62-1.34)	0.93 (0.63-1.37)	-	-	-	-	-	-

HD Score	-	-	-	-	1.27 (0.84-1.94)	1.26 (0.83-1.93)	-	-	-
PCA Score	-	-	-	-	-	-	-	0.97 (0.64-1.47)	0.98 (0.65-1.48)

Genetic model: age, age^2, sex, follow up years and ob-GRS. Lifestyle model: age, age^2, sex, FFQ type, follow up years, education, smoking status, alcohol intake, physical activity and diet scores. Combined model: Lifestyle model + ob-GRS

Significant values are marked in bold

Smoking status: non-smokers vs current smokers; education: school vs university education; physical activity: inactive vs active; alcohol intake: 1st vs 4th quartiles; ob-GRS: 1st vs 4th quartiles; NNR Score: 1st vs 4th quartiles; HD Score: 1st vs 4th quartiles; PCA Score: 1st vs 4th quartiles.

b) Type 2 Diabetes

Variables	Regression model (OR, 95% CI)								
	**NNR Score**			**HD Score**			**PCA Score**		
	**Genetic** (n= 2,017)	**Lifestyle** (n= 2,017)	**Combined** (n= 2,017)	**Genetic** (n= 2,087)	**Lifestyle** (n= 2,087)	**Combined** (n= 2,087)	**Genetic** (n= 2,017)	**Lifestyle** (n= 2,017)	**Combined** (n= 2,017)
Smoking status	-	**1.59** (1.06-2.37)	**1.55** (1.04-2.32)	-	**1.55** (1.05-2.30)	**1.53** (1.03-2.27)	-	**1.66** (1.11-2.48)	**1.62** (1.08-2.43)
Education	-	**0.57** (0.34-0.96)	**0.58** (0.34-0.98)	-	**0.56** (0.34-0.93)	**0.57** (0.34-0.95)	-	**0.55** (0.33-0.92)	**0.55** (0.33-0.94)
Alcohol intake	-	0.87 (0.52-1.45)	0.86 (0.51-1.44)	-	0.93 (0.56-1.53)	0.91 (0.55-1.51)	-	0.86 (0.52-1.44)	0.85 (0.51-1.43)
Physical activity	-	**0.51** (0.33-0.80)	**0.51** (0.33-0.79)	-	**0.54** (0.35-0.83)	**0.53** (0.35-0.82)	-	**0.49** (0.32-0.76)	**0.48** (0.31-0.75)
T2d-GRS	**1.84** (1.16-2.92)	-	**1.84** (1.15-2.94)	**1.9** (1.20-3.00)	-	**1.9** (1.20-3.02)	**1.84** (1.16-2.92)	-	**1.82** (1.14-2.91)
NNR Score	-	1.00 (0.63-1.59)	0.99 (0.62-1.57)	-	-		-	-	-
HD Score	-	-	-	-	1.26 (0.77-2.06)	1.22 (0.74-2.01)	-	-	-

PCA Score	-	-	-	-	-	-	-	**0.50** (0.31-0.83)	**0.52** (0.31-0.85)

Genetic model: age, age^2, sex, fasting status, follow up years and t2d-GRS. Lifestyle model: age, age^2, sex, fasting status, FFQ type, follow up years, education, smoking status, alcohol intake, physical activity and diet scores. Combined model: lifestyle model + t2d-GRS

Significant values are marked in bold

Smoking status: non-smokers vs current smokers; education: school vs university education; physical activity: inactive vs active; alcohol intake: 1st vs 4th quartile; t2d-GRS: 1st vs 4th quartile; NNR Score: 1st vs 4th quartile; HD Score: 1st vs 4th quartile; PCA Score: 1st vs 4th quartile

c) IFG

Variables	Regression model (OR, 95% CI)								
	NNR Score			HD Score			PCA Score		
	Genetic (n= 2,778)	Lifestyle (n= 2,778)	Combined (n= 2,778)	Genetic (n= 2,882)	Lifestyle (n= 2,882)	Combined (n= 2,882)	Genetic (n= 2,778)	Lifestyle (n= 2,778)	Combined (n= 2,778)
Smoking status	-	1.17 (0.91-1.50)	1.18 (0.91-1.51)	-	1.16 (0.91-1.48)	1.16 (0.91-1.49)	-	1.16 (0.91-1.49)	1.17 (0.91-1.50)
Education	-	0.84 (0.63-1.12)	0.85 (0.63-1.14)	-	0.89 (0.66-1.18)	0.90 (0.68-1.21)	-	0.83 (0.62-1.12)	0.85 (0.63-1.14)
Alcohol intake	-	1.16 (0.85-1.59)	1.15 (0.83-1.57)	-	1.18 (0.87-1.61)	1.16 (0.85-1.58)	-	1.16 (0.84-1.59)	1.14 (0.83-1.57)
Physical activity	-	0.92 (0.73-1.15)	0.93 (0.74-1.17)	-	0.93 (0.74-1.16)	0.94 (0.75-1.18)	-	0.91 (0.73-1.15)	0.93 (0.74-1.17)
fg-GRS	**1.67** (1.25-2.24)	-	**1.66** (1.24-2.23)	**1.73** (1.30-2.31)	-	**1.72** (1.29-2.30)	**1.67** (1.25-2.24)	-	**1.66** (1.24-2.23)
NNR Score	-	1.01 (0.77-1.34)	1.01 (0.76-1.33)	-	-	-	-	-	-
HD Score	-	-	-	-	0.87 (0.64-1.17)	0.87 (0.64-1.17)	-	-	-

PCA Score	-	-	-	-	-	-	-	1.09 (0.81-1.47)	1.10 (0.82-1.48)

Genetic model: age, age^2, sex, fasting status, follow up years and fg-GRS. Lifestyle model: age, age^2, sex, fasting status, FFQ type, follow up years, education, smoking status, alcohol intake, physical activity and diet scores. Combined model: Lifestyle model + fg-GRS

Significant values are marked in bold

Smoking status: non-smokers vs current smokers; education: school vs university education; physical activity: inactive vs active; alcohol intake: 1st vs 4th quartile; fg-GRS: 1st vs 4th quartile; NNR Score: 1st vs 4th quartile; HD Score: 1st vs 4th quartile; PCA Score: 1st vs 4th quartile

d) IGT

Variables	Regression model (OR, 95% CI)								
	NNR Score			HD Score			PCA Score		
	Genetic (n= 2,420)	Lifestyle (n= 2,420)	Combined (n= 2,420)	Genetic (n= 2,509)	Lifestyle (n= 2,509)	Combined (n= 2,509)	Genetic (n= 2,420)	Lifestyle (n= 2,420)	Combined (n= 2,420)
Smoking status	-	0.92 (0.72-1.18)	0.91 (0.71-1.17)	-	0.91 (0.71-1.16)	0.90 (0.70-1.15)	-	0.94 (0.73-1.21)	0.93 (0.72-1.20)
Education	-	0.79 (0.59-1.05)	0.79 (0.59-1.05)	-	0.80 (0.60-1.06)	0.79 (0.59-1.05)	-	0.78 (0.58-1.04)	0.77 (0.58-1.03)
Alcohol intake	-	1.26 (0.92-1.71)	1.26 (0.93-1.72)	-	1.27 (0.94-1.72)	1.27 (0.94-1.73)	-	1.28 (0.94-1.75)	1.29 (0.95-1.76)
Physical activity	-	**0.69** (0.55-0.86)	**0.69** (0.55-0.86)	-	**0.74** (0.59-0.92)	**0.74** (0.59-0.93)	-	**0.67** (0.54-0.85)	**0.67** (0.54-0.85)
2hg-GRS	**1.44** (1.11-1.87)	-	**1.46** (1.13-1.90)	**1.45** (1.12-1.87)	-	**1.46** (1.13-1.89)	**1.44** (1.11-1.87)	-	**1.46** (1.13-1.90)
NNR Score	-	1.05 (0.80-1.39)	1.05 (0.80-1.38)	-	-	-	-	-	-
HD Score	-	-	-	-	0.94 (0.71-1.26)	0.94 (0.71-1.26)	-	-	-

PCA Score	-	-	-	-	-	-	-	**0.71** (0.53-0.95)	**0.71** (0.53-0.95)

Genetic model: age, age^2, sex, fasting status, follow up years and 2hg-GRS. Lifestyle model: age, age^2, sex, fasting status, FFQ type, follow up years, education, smoking status, alcohol intake, physical activity and diet scores. Combined model: Lifestyle model + 2hg-GRS

Significant values are marked in bold

Smoking status: non-smookers vs current smokers; education: school vs university education; physical activity: inactive vs active; alcohol intake: 1st vs 4th quartile; 2hg-GRS: 1st vs 4th quartile; NNR Score: 1st vs 4th quartile; HD Score: 1st vs 4th quartile; PCA Score: 1st vs 4th quartile.
